# Measuring the Time to Deterioration for Health-Related Quality of Life in Patients With Metastatic Breast Cancer Using a Web-Based Monitoring Application: Longitudinal Cohort Study

**DOI:** 10.2196/25776

**Published:** 2021-10-12

**Authors:** Katharina Brusniak, Manuel Feisst, Linda Sebesteny, Andreas Hartkopf, Joachim Graf, Tobias Engler, Andreas Schneeweiss, Markus Wallwiener, Thomas Maximilian Deutsch

**Affiliations:** 1 Department of Gynecology and Obstetrics University Hospital Heidelberg Heidelberg Germany; 2 Institute of Medical Biometry and Informatics University of Heidelberg Heidelberg Germany; 3 Department of Women’s Health University Hospital Tübingen Tübingen Germany; 4 Department of Midwifery Science Institute for Health Sciences University Hospital Tübingen Tübingen Germany; 5 Department of Medical Oncology National Center for Tumor Diseases Heidelberg Germany; 6 German Cancer Research Center Heidelberg Germany

**Keywords:** eHealth, breast cancer, health-related quality of life, quality of life, time to deterioration, EQ-VAS, EQ-5D-5L, EORTC QLQ-C30

## Abstract

**Background:**

Health-related quality of life (HRQoL) is used to evaluate the treatment of metastatic breast cancer. In a long-term therapy setting, HRQoL can be used as an important benchmark for treatment success. With the help of digital apps, HRQoL monitoring can be extended to more remote areas and be administered on a more frequent basis.

**Objective:**

This study aims to evaluate 3 common HRQoL questionnaires in metastasized breast cancer in terms of TTD in a digital, web-based setting. We further aim to examine the development of the HRQoL in different systemic treatment groups in each of these evaluation instruments.

**Methods:**

A total of 192 patients with metastatic breast cancer were analyzed in this bicentric prospective online cohort study at two German university hospitals. Patients completed questionnaires on HRQoL (EuroQol Visual Analog Scale [EQ-VAS], EuroQol 5 Dimension 5 Level [EQ-5D-5L], European Organization for Research and Treatment of Cancer Quality of Life Questionnaire–Core 30 item [EORTC QLQ-C30]) via an online platform over a 6-month period. Treatment schedules and medical history were retrieved from medical records. Unadjusted Cox regression analysis on treatment-related factors was performed. We conducted subgroup analyses in regard to TTD events between different treatments.

**Results:**

The EQ-VAS showed a higher rate of deterioration after 8 weeks (84/179, 46.9%) than the EQ-5D-5L (47/163, 28.8%) and EORTC QLQ-C30 (65/176, 36.9%). Unadjusted Cox regression revealed significant connections between known metastases in the liver (*P*=.03, HR 1.64, 95% CI 1.06-2.52) and pleura (*P*=.04, HR 0.42, 95% CI 0.18-0.96) in the EQ-VAS. Significant relations between EQ-VAS events and single EQ-5D-5L items and the EQ-5D-5L summary score were demonstrated. All treatment groups significantly differed from the CDK4/6 inhibition subgroup in the EQ-VAS.

**Conclusions:**

Compared to the EQ-5D-5L and QLQ-C30, the EQ-VAS showed a higher rate of deterioration after 8 weeks. Significant connections to certain metastatic locations were only detected in the EQ-VAS. The EQ-VAS is capable of reflecting the distinctive HRQoL profiles of different systemic treatments as well as the different aspects of HRQoL presented in the EQ-5D-5L. TTD with the EQ-VAS is an adequate mean of examining longitudinal development of HRQoL among breast cancer patients.

## Introduction

Breast cancer is the most common cancer in women, with 1 in 8 women being affected throughout their lifetime [1]. Although there has been significant progress made both in detection and treatment, the prognosis of metastatic breast cancer remains poor. The more severe the disease, the more important palliative treatment options become that offer an acceptable health-related quality of life (HRQoL) while still providing the patient with individually optimized and life prolonging treatments [2]. There is a strong connection between HRQoL and factors such as progression of disease, progression-free survival, and the experience of adverse events during therapy [2-4]. In addition, HRQoL measurements can help with doctor-patient communication and can even be beneficial to the HRQoL itself when discussing the assessments with the physician [5]. Moreover, patients with fulfilled information needs or higher satisfaction with the received information may also display a higher degree of HRQoL [6].

Various factors can influence a patient’s HRQoL making it a variable that is both difficult to unify and to diversify. The concept can mean something different to every patient, leading to a variety of interpretative possibilities. Therefore, the concept of HRQoL bares the difficulty of objectifying its content for practical decision making in medical practice. Aspects that play into the concept of HRQoL in modern medicine can vary from independence, stage of disease, the amount and severity of drug side effects to even personal fulfillment. As diverse as the topic itself are the options of evaluating it [7]. In recent years an emphasis has been made on patient-reported outcomes (PRO) as a means of collecting HRQoL data. PROs are characterized by the fact that several validated questionnaires are used simultaneously for HRQoL measurement in order to balance the respective subjectivity [8].

A PRO is “a measurement based on a report that comes directly from the patient (ie, study subject) about the status of a patient’s health condition without amendment or interpretation of the patient’s response by a clinician or anyone else” [9]. They are an effective module in assessing a patient’s well-being using paper-based and digital data collection [10]. They are useful in identifying patient distress and assessing new therapeutic methods and can hence improve care [11,12]. A previous study also observed a benefit in overall survival for patients who self-reported their symptoms in an online setting [13]. However, PRO data depend on factors that may not be health-related or influenced by individual values or other passing momentary conditions [14]. In addition, practical aspects can influence HRQoL data collection. An overflow of long questionnaires can influence compliance and motivation [15,16]. Furthermore, several studies have reported poor compliance in long-term studies [17,18]. While the findings did not show conclusively if compliance was dependent on the questionnaire format (visual analog or categorical) [17], the chosen evaluative instrument can have an influence on people’s perception and adoption of it [7]. These issues play an important role when administering studies digitally, especially to a less technically inclined collective, such as older patients [19].

Therapeutic decision making, especially in palliative care, can depend on the patient’s reporting of their HRQoL. As data suggest that clinicians may underestimate or miss a large part of adverse effects, there is a need for more clarity in physicians’ evaluation of patient-reported content [20-23]. Changes and time to deterioration (TTD) in HRQoL have previously been used to further assess the benefits of cancer medication [24,25], again emphasizing the high potential of a differentiated evaluation of HRQoL assessments in cancer research. With metastatic breast cancer patients usually being treated for a longer period of time at the same care center, detecting change in patient-reported HRQoL presents a type of measurement that allows for long-term HRQoL screening in addition to isolated assessments. Exploring the longitudinal development of HRQoL with the TTD method may help uncover influential factors on HRQoL as well as predictive capabilities of such measurements [26]. The introduction of digital monitoring systems in the area of HRQoL offers new possibilities in reaching out to patients struggling with the effects of metastatic breast cancer and extend medical care to remote areas. However, the digital application of a longitudinal measurement system needs to be evaluated in terms of effectiveness, acceptance and presentation. Low compliance can be a challenge in longitudinal digital studies [18], and aspects concerning patients’ expectation regarding quality of life such as response shift can influence the TTD [27].

This study aimed to evaluate 3 common HRQoL questionnaires (EuroQol Visual Analog Scale [EQ-VAS], EuroQol 5 Dimension 5 Level [EQ-5D-5L], European Organization for Research and Treatment of Cancer Quality of Life Questionnaire–Core 30 item [EORTC QLQ-C30]) in a sample of women with metastasized breast cancer in terms of TTD in a digital, web-based setting. We further examined the development of the HRQoL in different systemic treatment groups.

## Methods

### Study Design and Sample

**Figure 1:**
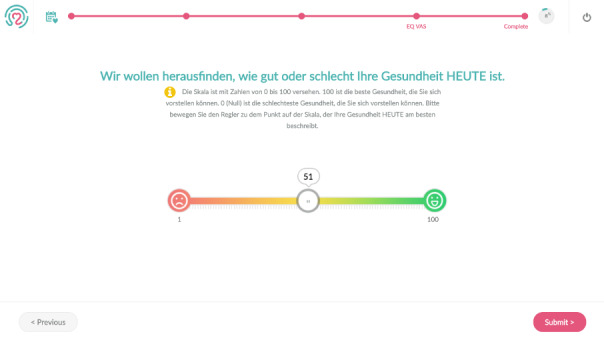
Example of an assessment section on the Patient-informiert-interaktiv-Arzt portal.

The PEPPER study (Patient Engagement Pilotstudie Mammakarzinom-individualisierte und Ressourcen-effiziente Patient-Reported Outcomes Erfassung durch digitale Therapieunterstuetzungssysteme) was conducted from December 2016 to August 2019 at two German university hospitals (University Hospitals of Heidelberg and Tübingen). It was designed as a bicentric prospective cohort study collecting longitudinal information on HRQoL, physical symptoms, and PROs of metastatic breast cancer patients via the online platform PiiA (Patient-informiert-interaktiv-Arzt, Figure 1) over a 6-month period. The assessments were scheduled weekly for the first 8 weeks of the cohort study and 4 times monthly for the last 4 months (see Table 1). The digital assessment of QoL allowed for evaluation not bound to treatment schedule and the inclusion of patients not living in close proximity to the care center. Participants were identified through a screening process of their medical history and then approached at their next scheduled appointment. Criteria of eligibility were ≥18 years of age, a sufficient level of the German language, metastatic breast cancer in progressive or stable state of disease undergoing any form of systemic therapy, patients with therapy change, active enrollment in the PRAEGNANT study (a German metastatic breast cancer registry network), and written consent. Exclusion criteria covered patients who were not eligible for observation due to severe comorbidities or unavailability according to the treating physician, patients who were not able to handle a tablet computer or were unable to write as well as patients who were not able to understand the nature and extent of the trial and the procedures required.

The patients assessed for eligibility were radiologically assessed for disease progression every 3 months until death or loss to follow-up using the Response Evaluation Criteria in Solid Tumors [28]. The patients assessed for eligibility were divided into 2 subgroups—patients with stable disease or partial response and those with early progressive disease at the first trimonthly follow-up evaluation.

Upon confirmation of participation, patients were asked to complete the baseline visit on-site on a tablet provided by the staff. Skilled staff was available throughout the baseline visit in person and via email during the entire study period to provide technical support. Further parts of the study were completed on their preferred device at home. Participants of the study were reminded of upcoming or uncompleted visits via email or telephone. The study was conducted in German. Ethics approval was granted by the ethical committees of the University of Heidelberg (S-598/2016) and Tübingen (191/2017BO2).

**Table 1:** Implementation of questionnaires.

Visit	Baseline	1	2	3	4	5	6	7	8	9	10	11	12
Week	0	1	2	3	4	5	6	7	8	12	16	20	24
EQ-VAS^a^	✓	✓	✓	✓	✓	✓	✓	✓	✓	✓	✓	✓	✓
EQ-5D-5L^b^	✓	✓	✓	✓	✓	✓	✓	✓	✓	✓	✓	✓	✓
EORTC QLQ-C30^c^	✓	—^d^	—	—	✓	—	—	—	✓	✓	✓	✓	✓

^a^EQ-VAS: EuroQol Visual Analog Scale.

^b^EQ-5D-5L: EuroQol 5 Dimension 5 Level.

^c^EORTC QLQ-C30: European Organization for Research and Treatment of Cancer Quality of Life Questionnaire-Core 30 item.

^d^Not applicable.

### Quantitative Data Collection and Questionnaires

Sociodemographic data was gathered at baseline via the online platform PiiA. In addition, treatment regiments and medical history were retrieved by analyzing medical records of the particular university hospital. To evaluate the QoL of the patients, 3 assessment instruments were used in this study.

We administered 3 common HRQoL questionnaires (EQ-VAS, EQ-5D-5L, EORTC QLQ-C30) over a 6-month period (see Table 1). A TTD event is defined as the decline in HRQoL score in the respective questionnaire score by the corresponding minimally important difference (MID) in comparison to the baseline score.

The EQ-VAS is a global self-evaluation of the state of health on a visual analog scale from 0 (worst imaginable state of health) to 100 (best imaginable state of health). It thereby offers a global and momentary insight into the patients’ overall self-reported well-being. The EQ-VAS can be administered as part of the EQ-5D questionnaire [29,30]. A difference of ≥7 points was the MID for deterioration detection, which has previously been established in similar studies [24,25,31,32].

The EQ-5D-5L is a validated questionnaire consisting of 5 questions, each with 5 options, encompassing aspects such as mobility and self-reliance as parts of its HRQoL definition [33]. The EQ-5D-5L is a validated instrument in assessing HRQoL in German [29,34] and has shown to be of use in detecting changes in the state of health of breast cancer patients [35]. The EQ-5D-5L can be summarized using a score ranking from <0 (worst possible HRQoL) to 1 (best possible HRQoL) [33]. A decrease in ≥0.08 points was regarded as a MID for deterioration as described previously [24,25,31,32]. The average completion time for the EQ-5D-5L ranges from 25 to 75 seconds, while the EQ-VAS can be answered in just 5 to 15 seconds.

The EORTC QLQ-C30 constitutes a more detailed questionnaire in regard to HRQoL and is a valid tool in measuring the HRQoL in cancer patients [36]. Consisting of 30 items, the EORTC QLQ-C30 encompasses 5 questions about self-reliance in everyday situations, 23 questions about physical complaints and their impact on HRQoL and social interactions on 4-point Likert scales as well as two global items on the HRQoL and state of health, each on a 7-point Likert scale. The average time to completion of this questionnaire is estimated to range from 150 to 450 seconds. The QLQ-C30 is summed up using a summary score [37]. The questionnaire has previously been found to be a valid instrument in assessing HRQoL in breast cancer patients via an eHealth medium [38]. In accordance with similar studies, a decline of ≥10 points was regarded as deterioration [24,31,39-41]. The pattern, in which questionnaires were implemented in the study, is depicted in Table 1.

### Treatment Line Grouping

Data about their current treatment regime was assembled from the participants’ medical history. The various lines of treatment were divided into the following 4 groups: cyclin-dependent kinase (CDK) 4/6 inhibition therapy (including any form of endocrine therapy in combination with a CDK4/6 inhibitor), human epidermal growth factor receptor 2 (HER2)-targeted therapy (including trastuzumab, pertuzumab, trastuzumab emtansine, and lapatinib alone or in combination with chemotherapy), chemotherapy (intravenous or oral) alone, and endocrine therapy alone.

### Statistical Analysis

We used the programming language R (version 3.6.1, R Foundation for Statistical Computing) for all analyses [42]. Socioeconomic characteristics, questionnaire data, and treatment schedules were first described descriptively using absolute and relative frequencies, means, and standard deviations.

TTD was defined as time to the first clinically meaningful deterioration in the respective HRQoL assessment tool and was illustrated using Kaplan-Meier plots. Furthermore, univariable, unadjusted Cox regression was applied to examine the influence of state of disease and similar characteristics on the TTD for all questionnaires. Moreover, we examined the aforementioned systemic treatment groups as to their TTD events for the EQ-VAS and the EQ-5D-5L using unadjusted Cox regression. Furthermore, predetermined systemic treatment groups within each HRQoL questionnaire were compared using linear mixed models.

Thereupon, EQ-VAS scores were compared to the different questions of the EQ-5D-5L as well as to the EQ-5D-5L summary score. For the patients who experienced a TTD event in the EQ-VAS, the difference of the values between the time of the event and the baseline visit in the respected EQ-5D-5L item were compared by applying the 1-sample Wilcoxon signed-rank test. Thereafter, this difference was compared to the differences of patients without a TTD event using the 2-sample Wilcoxon rank-sum test. In all analyses, *P*<.05 (2-tailed) was considered indicative of statistically significant differences.

## Results

### Sociodemographic Characteristics and State of Disease

A total of 192 patients with metastatic breast cancer were analyzed in this bicentric prospective online cohort study at two German university hospitals. During the first 8 weeks of the study, 21.9% (42/192) of participants completed every visit with a satisfactory completion rate of ≥80% showing a considerable loss of patients during follow-up in the overall study. However, the percentage of completed questionnaires after 8 weeks in comparison to baseline was higher with 62.7% (104/166) for the EQ-VAS, 73.2% (82/112) for the EQ-5D, and 62.4% (103/165) for the QLQ-C30. The number of completed questionnaires for each visit are included in Multimedia Appendix 1. The sociodemographic characteristics of this collective are shown in Table 2. The average age at study inclusion was 54.3 years. A total of 49.5% (95/192, 25 missing) of patients had a high education level (university entrance qualification or higher), and 69.8% (134/192, 25 missing) received public health insurance.

**Table 2:** Sociodemographic characteristics (n=192).

Characteristic			Value
Age at study inclusion (years), mean (SD)			54.3 (10.1)
Age at primary diagnosis (years), mean (SD)			47.3 (10.0)
**Education, n (%)**			
	University entrance qualification or higher	95 (49.5)	
	Lower than university entrance	72 (37.5)	
	Missing	25 (13.0)	
**Health insurance, n (%)**			
	Public	134 (69.8)	
	Private	33 (17.2)	
	Missing	25 (13.0)	
**Marital status, n (%)**			
	Married/in a relationship	142 (74.0)	
	Not married/in a relationship	23 (12.0)	
	Missing	27 (14.1)	
**Children, n (%)**			
	Yes	128 (66.7)	
	No	39 (20.3)	
	Missing	25 (13.0)	

The mean age of initial diagnosis was 47.3 years. The average duration between initial diagnosis and study inclusion was 66.6 months. A total of 29.7% (57/192, 57 missing) of patients were already in metastatic stage at initial diagnosis of breast cancer. Further information on the metastatic situation at study enrollment and state of disease of the primary tumor according to TNM classifications can be seen in Table 3.

The median number of different treatment regiments before inclusion was 3 (range 0-13, Q1-Q3 2-4) and on average patients received 1 (0-10, 1-2) different chemotherapeutic treatment lines prior to enrollment in the study. Within the first 3 months of study participation, 46 patients (46/192, 24.0%, 11 missing) were diagnosed with disease progression and 21 patients (21/192, 10.9%, 10 missing) experienced a change in treatment. The systemic treatment line patients followed throughout this period is shown in Table 3.

**Table 3:** State of disease and treatment regiments.

Characteristic			Value
Difference between initial diagnosis of breast cancer and study inclusion (months), median (Q1-Q3)			66.6 (29.4-127.4)
Difference between initial diagnosis of breast cancer metastases and study inclusion (months), median (Q1-Q3)			21.5 (6.8-40.1)
**Characteristics of primary tumor (TNM classification), n (%)**			
	**c/y/pT^a^PT^b^**		
		0	7 (3.6)
		1	46 (24.0)
		2	60 (31.3)
		3	15 (7.8)
		4	7 (3.6)
		Other or N/A	57 (29.7)
	**c/y/pN^c^PT**		
		+	35 (18.2)
		0	23 (12.0)
		1	32 (16.7)
		2	13 (6.8)
		3	6 (3.1)
		Other or N/A	83 (43.2)
	**M^d^PT**		
		1	57 (29.7)
		0	78 (40.6)
		N/A	57(29.7)
**Breast cancer subtype of primary tumor, n (%)**			
	Hormone receptor positive + HER2^e^ neu negative		101 (52.6)
	HER2 neu positive		63 (32.8)
	Triple negative		14 (7.3)
	N/A		14 (7.3)
**Metastases diagnosed at study inclusion, n (%)**			
	Brain		6 (3.1)
	Lymph nodes		53 (27.6)
	Bone		108 (56.3)
	Lung		55 (28.6)
	Pleura		20 (10.4)
	Liver		66 (34.4)
	Peritoneum		9 (4.7)
	Skin		10 (5.2)
	Other		15 (7.8)
	N/A		4 (2.1)
**Previous treatment regiments before study inclusion (Q1-Q3)**			
	Number of treatment regiments, median		3 (2-4)
	Number of chemotherapeutic treatment lines, median		1 (1-2)
**Systemic treatment groups during study period, n (%)**			
	CDK^f^ 4/6 inhibitors +/– endocrine therapy		41 (21.4)
	Chemotherapy		62 (32.3)
	Endocrine therapy		18 (9.4)
	HER2-targeted therapy		54 (28.1)
	N/A		17 (8.9)

^a^c/y/pT: Clinical/after neoadjuvant therapy/pathologic classification of tumor extent and size.

^b^PT: Primary tumor.

^c^c/y/pN: Clinical/after neoadjuvant therapy/pathologic classification of regional lymph node involvement.

^d^M: Metastatic spread.

^e^HER2: human epidermal growth factor receptor 2.

^f^CDK: cyclin-dependent kinase.

### Questionnaire Data

**Figure 2:**
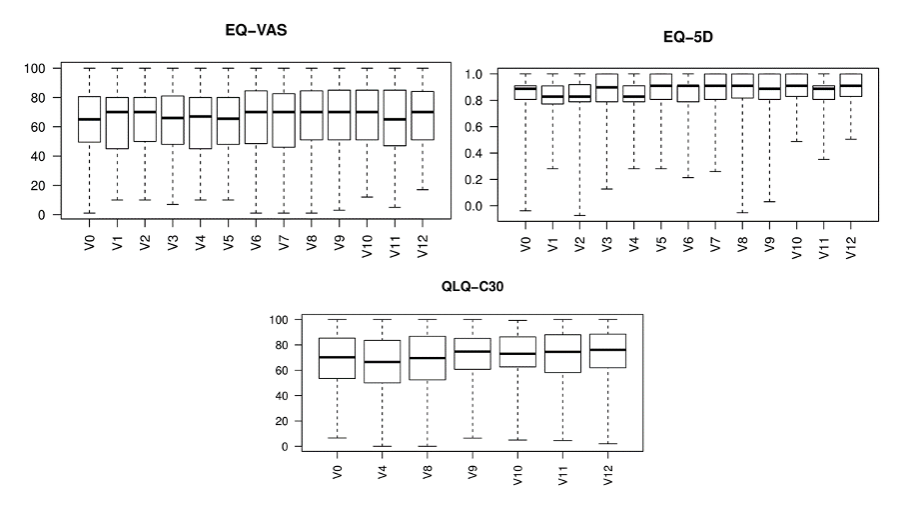
Box plots representing (a) EQ-VAS results at baseline and 12 visits, (b) EQ-5D-5L results at baseline and 12 visits, and (c) EORTC-QLQ-C30 results at baseline and 6 visits. EORTC QLQ-C30: European Organization for Research and Treatment of Cancer Quality of Life Questionnaire-Core 30 item; EQ-5D-5L: EuroQol 5 Dimension 5 Level; EQ-VAS: EuroQol Visual Analog Scale.

Figure 2 shows the overall state of health at the different visits. On average, patients reported a health status in the upper half of the possible range in each of the questionnaires and at all visits. Furthermore, the differences observed throughout the 6-month study period are fairly small in all questionnaires, indicating a low degree of change in HRQoL during the study period. The EQ-VAS consistently showed a higher variance than the other questionnaires during the entire study period.

### TTD With Regression Results

The rate of deterioration (number of patients with deterioration divided by the total number of patients) amounted to 0.47 in the EQ-VAS (84/179), representing the highest rate of TTD events in our sample with an average TTD of 8 weeks. We could identify a rate of deterioration of 0.29 (47/163) in the EQ-5D-5L and 0.37 (65/176) in the QLQ-C30.

**Figure 3:**
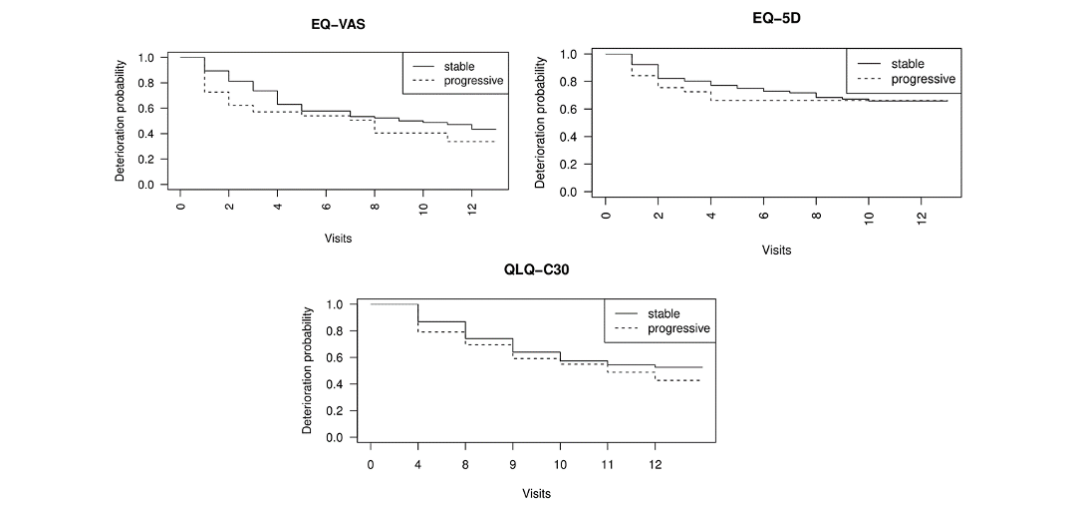
Kaplan-Meier estimation for stable and progressive state of disease representing (a) EQ-VAS, (b) EQ-5D-5L, and (c) EORTC-QLQ-C30. EORTC QLQ-C30: European Organization for Research and Treatment of Cancer Quality of Life Questionnaire-Core 30 item; EQ-5D-5L: EuroQol 5 Dimension 5 Level; EQ-VAS: EuroQol Visual Analog Scale.

Univariate Cox regression analysis on pathologic and treatment-related factors showed a connection between known metastases in the liver (*P*=.03) and pleura (*P*=.04) at the time of study inclusion and deterioration, as well as a vague link to the clinical diagnoses of disease progression within the first 3 months of the study in the EQ-VAS (*P*=.11). As can be seen in Figure 3, patients with diagnosed disease progression (hazard ratio [HR] 1.48) showed a higher rate of TTD events in the EQ-VAS than in case of no progression with a nonsignificant *P* value (*P*=.11), as seen in Figure 3. For the other questionnaires, we could not detect a link between the reviewed criteria and deterioration. The results of the univariate Cox regression analysis can be found in Table 4. A univariate Cox regression analysis with results adjusted for age and progression can be found in Multimedia Appendix 1.

**Table 4:** Univariate Cox regression analysis.

Variable			EQ-VAS^a^				EQ-5D-5L^b^					EORTC QLQ-C30^c^		
			Hazard ratio (95% CI)		*P* value		Hazard ratio (95% CI)		*P* value		Hazard ratio (95% CI)			*P* value
Age			0.99 (0.97-1.02)		.583		0.98 (0.95-1.01)		.20		1.00 (0.98-1.03)			.83
**Metastasis**														
	Brain	1.71 (0.74-3.94)		.21		0.32 (0.04-2.31)		.26		1.16 (0.42-3.2)			.78	
	Lymph nodes	1.20 (0.77-1.87)		.42		1.42 (0.79-2.54)		.23		0.95 (0.56-1.63)			.86	
	Bone	0.83 (0.54-1.29)		.41		0.85 (0.47-1.53)		.59		1.4 (0.81-2.43)			.22	
	Lung	1.09 (0.69-1.69)		.71		0.73 (0.39-1.37)		.33		0.97 (0.57-1.65)			.91	
	Pleura	0.42 (0.18-0.96)		.04		0.43 (0.13-1.39)		.16		0.90 (0.41-1.98)			.79	
	Liver	1.64 (1.06-2.52)		.03		1.67 (0.94-2.99)		.08		0.87 (0.51-1.47)			.61	
	Peritoneum	0.96 (0.35-2.63)		.94		0.78 (0.19-3.2)		.73		1.39 (0.5-3.84)			.52	
	Skin	0.55 (0.2-1.52)		.25		0.82 (0.25-2.65)		.74		0.41 (0.1-1.7)			.22	
	Other	0.67 (0.32-1.39)		.28		0.73 (0.26-2.04)		.55		1.79 (0.93-3.46)			.08	
Progression			1.48 (0.91-2.37)		.11		1.15 (0.60-2.23)		.34		1.05 (0.58-1.88)			.88
**Systemic group**														
	CDK^d^ 4/6 inhibitors +/– endocrine therapy	Reference		Ref		Ref		Ref		Ref			Ref	
	Chemotherapy	1.72 (0.77-3.85)		.19		1.48 (0.41-5.32)		.55		0.43 (0.2-0.94)			.03	
	Endocrine therapy	2.29 (0.93-5.65)		.07		0.69 (0.11-4.21)		.69		0.60 (0.25-1.5)			.29	
	HER2^e^-targeted therapy	1.7 (0.76-3.83)		.20		1.42 (0.38-5.4)		.60		0.54 (0.25-1.15)			.11	

^a^EQ-VAS: EuroQol Visual Analog Scale.

^b^EQ-5D-5L: EuroQol 5 Dimension 5 Level.

^c^EORTC QLQ-C30: European Organization for Research and Treatment of Cancer Quality of Life Questionnaire–Core 30 item.

^d^CDK: cyclin-dependent kinase.

^e^HER2: human epidermal growth factor receptor 2.

### Systemic Treatment Groups

We divided the patients into 4 groups according to the treatment that they received during the first 3 months of the study. We then proceeded to use Cox regression to compare the subgroups with each other in terms of the TTD. This revealed a difference between CDK4/6 inhibitor therapy and mere endocrine therapy in the EQ-VAS (*P*=.07) and between CDK4/6 inhibitor therapy and chemotherapy in the QLQ-C30 (*P*=.03; see Table 4).

Using a linear mixed model, we proceeded to compare the predetermined systemic treatment groups within each HRQoL questionnaire. For the EQ-VAS and EQ-5D-5L, a significant difference between treatment groups could be detected. In the EQ-VAS, all treatment groups showed a significant difference in comparison to CDK4/6 inhibitor therapy during the examination period (see Table 5). A similar difference showed in our analyses of these subgroups using the QLQ-C30 summary score. An increase in the difference of EQ-VAS values in comparison to baseline is visible for patients receiving CDK4/6 inhibitors. For the EQ-5D-5L, a significant difference between patients receiving chemotherapy and HER2-targeted therapy could be encountered. All results of the subgroup analysis can be examined in Table 5.

**Table 5:** Linear mixed model and post hoc analysis results for therapeutic subgroup comparison (cyclin-dependent kinase 4/6 inhibitors +/– endocrine therapy = group 1, chemotherapy = group 2, endocrine therapy = group 3, human epidermal growth factor receptor 2-targeted therapy = group 4); scale of the respective tool in brackets.

Group comparison	EQ-VAS^a^ (0-100)		EQ-5D-5L^b^ (0-1)			EORTC QLQ-C30^c^ (0-100)	
	Estimate	*P* value	Estimate	*P* value	Estimate		*P* value
Overall	—^d^	<.001	—	.002	—		.048
2-1	–14.41	<.001	–0.06	.03	8.91		.04
3-1	–12.62	<.001	–0.26	.79	1.74		.97
4-1	–10.58	<.001	0.01	.99	3.37		.75
3-2	1.80	.81	0.04	.47	–7.17		.23
4-2	3.83	.09	0.07	.002	–5.54		.29
4-3	2.03	.75	0.03	.58	1.63		.97

^a^EQ-VAS: EuroQol Visual Analog Scale.

^b^EQ-5D-5L: EuroQol 5 Dimension 5 Level.

^c^EORTC QLQ-C30: European Organization for Research and Treatment of Cancer Quality of Life Questionnaire–Core 30 item.

^d^Not applicable.

### Event Comparison Between the EQ-VAS and the EQ-5D-5L and Patients Without TTD Events

For each patient who showed deterioration in the EQ-VAS, a Wilcoxon signed-rank test with continuity correction was conducted to examine whether significant differences in singular questions and the summary score of the EQ-5D-5L could be detected. In Table 6 it can be seen that for several EQ-5D-5L items such a significant relation could be registered. Thereupon, a 2-sample Wilcoxon rank-sum test was performed comparing the deteriorating patients to the rest of the sample group to further differentiate between significant subgroup and collective deterioration. The results are depicted in Table 6.

**Table 6:** Results of the Wilcoxon signed rank test and the 2-sample Wilcoxon rank-sum test.

EQ-5D-5L^a^ scale	Event: baseline			Event: remaining time steps	
	Mean difference (CI)	*P* value^b^	Mean difference (CI)		*P* value^c^
Mobility	0.28 (0.09 to 0.48)	.005	0.34 (0.14 to 0.54)		.001
Selfcare	0.15 (–0.01 to 0.31)	.06	0.12 (–0.03 to 0.29)		.12
Activities	0.26 (0.05 to 0.47)	.02	0.29 (0.07 to 0.51)		.01
Pain	0.26 (0.07 to 0.45)	.009	0.27 (0.07 to 0.47)		.008
Anxiety	0.26 (–0.04 to 0.56)	.09	0.23 (–0.07 to 0.54)		.13
Summary score	–0.06 (–0.11 to –0.01)	.02	–0.06 (–0.11 to –0.01)		.03

^a^EQ-5D-5L: EuroQol 5 Dimension 5 Level.

^b^Wilcoxon signed-rank text.

^c^Wilcoxon rank-sum test.

## Discussion

### Objective and Main Findings

In this study, we aimed to examine the longitudinal development of HRQoL using the TTD method in 3 different HRQoL questionnaires among breast cancer patients. We also applied Cox regression to determine possible influencing factors and used the Wilcoxon signed-rank test and the 2-sample Wilcoxon rank-sum test to distinguish our findings further. We then compared common systemic treatment groups in breast cancer treatment to emphasize our results. Mainly, we found the EQ-VAS showing a higher rate of deterioration than the other questionnaires in the same collective. Furthermore, in our sample the EQ-VAS offered a higher variance than the other questionnaires, allowing for more distinction between higher and lower outcome patients than the other instruments. A TTD event in the EQ-VAS also shows relations to disease related determinants as well as clear differentiation both individually between the EQ-VAS and the EQ-5D-5L items and from patients who did not experience a TTD event.

### TTD With Regression Results

The highest rate of deterioration using TTD method could be detected in the EQ-VAS, a visual analog scale. The MID that were used for deterioration detection have been previously used in other studies [24,25,31,32,39-41]. Nonetheless, it should be noted that the sample size for the EQ-VAS was bigger than for the other questionnaires, especially the EQ-5D-5L. It has been described that long questionnaires can result in lower compliance [16]. This might be explained by the length and timing of the other questionnaires: the other 2 instruments are more extensive and the QLQ-C30 was only included on a monthly basis. Implementing the QLQ-C30 on a monthly rather than a weekly basis was an effort to ensure compliance and motivation as this questionnaire is much longer than the other assessments and as this analysis only constitutes a secondary aim of this study. However, this may have resulted in patient loss within the interval and fewer opportunities to apply the TTD method on this questionnaire. Apart from this, due to the small sample size and the limited HRQoL variance in all questionnaires, we did not perform tests to compare the precision of the questionnaires among each other. Therefore, it cannot be concluded that the EQ-VAS is advantageous in the longitudinal investigation of HRQoL compared to the other questionnaires examined. However, although overall completion rates deteriorated over time as expected, the EQ-VAS showed a higher completion rate than the EQ-5D-5L, which were both included in the visits on a weekly basis. Hence, it can be concluded that the EQ-VAS as a single visual analog scale with decisive wording offers an easy application of HRQoL monitoring in a digital setting.

Using univariate Cox regression analysis on the pathologic and treatment-related factors we discovered a link between metastases in the liver (*P*=.03) and pleura (*P*=.04) at the time of study inclusion and deterioration in HRQoL only in the EQ-VAS. As metastases in other organs result in further symptoms, a decrease of HRQoL in this state of disease is very plausible. Patients with progressive disease showed a tendency of a shortened TTD in the EQ-VAS (HR 1.48) when compared to the EQ-5D-5L (HR 1.15) and the QLQ-C30 (HR 1.05). This corresponds to previous research that describes a negative impact of disease progression on HRQoL [3]. This connection might show possible predictive capabilities of this method when using the EQ-VAS, as it may be more sensitive to disease progression than the other questionnaires.

### Event Comparison Between the EQ-VAS and EQ-5D-5L

With the EQ-5D-5L and EORTC QLQ-C30 showing less deterioration events in comparison to the EQ-VAS and no significant connections to the above-described factors, we proceeded to further investigate the significance of a TTD event in the EQ-VAS. To accomplish this, we first applied a Wilcoxon signed-rank test with continuity correction. We observed significant changes for patients with an EQ-VAS event in several EQ-5D-5L items. This indicates an internal consistency of deterioration in HRQoL for individuals with an EQ-VAS TTD event among the several different aspects of HRQoL presented in the EQ-5D-5L. Moreover, it offers an assurance that aspects of the HRQoL definition of the EQ-5D-5L are reflected in the open formulation of the EQ-VAS. As the analysis showed only a vague relation to the anxiety question of the EQ-5D-5L, it might suggest a capability of the EQ-VAS to better reflect physical rather than mental aspects of HRQoL in breast cancer patients. However, the EQ-VAS has previously been reported to show a lower score in patients with anxiety and depressive disorders in comparison to healthy participants [43]. Nonetheless, in this sample a TTD event in the EQ-VAS was more strongly reflected in physical aspects of the EQ-5D-5L.

Thereupon, we performed a 2-sample Wilcoxon rank-sum test to contrast patients who experienced a TTD event in the EQ-VAS with patients who experienced no TTD event by comparing their respective differences in the EQ-5D-5L. As these analyses were significant for most items and the overall score, a clear distinction of patients with a TTD event to the inconspicuous participants became apparent. These analyses show that TTD events did not occur randomly but show that patients with a TTD event in the EQ-VAS significantly differ from the rest of the study population. This further supports the EQ-VAS as a valid screening instrument to implement TTD for longitudinal HRQoL management.

### Systemic Treatment Groups

Cox regression analyses revealed a vague statistical difference between patients receiving CDK4/6 inhibitors and patients undergoing endocrine therapy alone. As other studies reported factors such as pain reduction and advantageous tumor response for patients receiving a CDK4/6 inhibitor in addition to endocrine therapy, our findings offer a plausible reflection of CDK4/6 inhibitors’ HRQoL profile [44,45]. Furthermore, the combination with CDK4/6 inhibitors and endocrine therapy has shown to be beneficial in regard to progression-free survival when compared to endocrine therapy alone, which in turn represents an important factor in HRQoL [3,46].

From further examination of the EQ-VAS score using a linear mixed model (Table 5), we again found that CDK4/6 inhibition therapy significantly differs from the other treatment groups. Patients receiving CDK4/6 inhibition therapy showed an overall positive difference to baseline in the EQ-VAS during the entire study period, whereas the other groups showed a steady or even declining level of HRQoL on the questionnaire. As it has previously been reported that patients under CDK4/6 inhibitors have a slower rate of deterioration in HRQoL and experience milder side effects, our findings are reinforced by previous research [47,48]. This again supports our finding that a longitudinal observation of HRQoL through the EQ-VAS questionnaire is an adequate mean of measurement for this variable.

Further investigation of the EQ-5D-5L uncovered a significant difference between patients under chemotherapy and patients receiving HER2-targeted therapy. It has previously been described that patients who receive a combination of HER2-targeted therapy and chemotherapy exhibit better HRQoL than patients who only receive chemotherapy [49,50]. It has also been reported that the addition of HER2-targeted medication to a chemotherapy schedule can result in the improvement of adverse effects [49]. As can be seen in Multimedia Appendix 1, both groups showed a greater variance in the EQ-5D-5L than the other groups. For the subgroup undergoing HER2-targeted therapy, several extreme outliers with a high positive difference to baseline contribute to the distinction of this group. On the other hand, the boxplots for the chemotherapy subgroup show a discrete tendency toward a reduction in HRQoL on the EQ-5D-5L, which complements previous research.

This subgroup analysis therefore consolidates the representativeness of both our sample and our finding that measuring the TTD can be an adequate method to observe HRQoL, especially with the EQ-VAS.

However, not all treatment groups were of equal size and not all of these groups showed an adequate retention rate in their assessments. Therefore, these findings must be interpreted with proper caution, but in the context of previous studies in this area still represent an important impulse of future research.

### Limitations

Our analysis is based on a relatively small sample size. This might result from poor compliance, length of questionnaire or technical difficulties which, when present, were quickly resolved by the staff [15,16].

In addition, we did not account for response shift (“a change in the meaning of one’s self evaluation of a target construct“ [51]) as this was a secondary aim of this study. However, there are studies that show that by not considering response shift, HRQoL levels can lead to misinterpretation [52,53]. We also defined TTD events in relation to the baseline score. When assessing HRQoL, using the time until definitive deterioration has also been suggested in a metastatic setting [54]. In accordance with previous research in the field of longitudinal HRQoL monitoring and per not accounting for response shift in our analyses, we decided to apply the TTD method using the baseline score as reference [24,25,27,31,41,54].

Therefore, more research is needed to consolidate our findings. Moreover, all questionnaires were administered digitally only. However, the equivalence of electronic and paper-based PRO measurements has previously been established [10]. Furthermore, we detected a rather high and steady level of well-being among all questionnaires in our descriptive analysis, which limits the variance of these findings. We only included patients with internet access at home, as per inclusion criteria. Hence, older patients who are not as technologically inclined were not eligible for participation. Therefore, with an average age of 54.3 years, our sample does not reflect the average age of breast cancer patients [55]. Furthermore, as Heidelberg and Tübingen reflect economically strong regions in Germany, our sample showed a higher percentage of private health insurance and higher education than the general public [56-58]. As private health insurance in Germany is only available if you have a higher income, it can be concluded that our sample shows a bias in regard to its socioeconomic profile [56]. In addition, the order of the questionnaires remained the same throughout the study and was not randomized.

### Conclusions

In comparison to the EQ-5D-5L and QLQ-C30, the EQ-VAS showed a higher rate of deterioration, significant connections between deterioration and certain locations of metastases, and a better discrimination between progressive and stable disease (HR 1.48). In addition, known differences in HRQoL profiles of various treatment regiments were reflected in the EQ-VAS. We suggest that using the TTD method with the EQ-VAS is an adequate means of examining longitudinal development of HRQoL among breast cancer patients in a digital setting and constitutes a reasonable addition to breast cancer therapy.

## Appendix

Multimedia Appendix 1 Supplemental tables and figure.

## Abbreviations

CDK: cyclin-dependent kinase
EORTC QLQ-C30: European Organization for Research and Treatment of Cancer Quality of Life Questionnaire-Core 30 item
EQ-5D-5L: EuroQol 5 Dimension 5 Level
EQ-VAS: EuroQol Visual Analog Scale
HER2: human epidermal growth factor receptor 2
HR: hazard ratio
HRQoL: health-related quality of life
MID: minimally important difference
PEPPER study: Patient Engagement Pilotstudie Mammakarzinom - individualisierte und Ressourcen-effiziente Patient-Reported Outcomes Erfassung durch digitale Therapieunterstuetzungssysteme
PiiA: Patient-informiert-interaktiv-Arzt
PRO: patient-reported outcome
TTD: time to deterioration
