# Supplementary material for: Measuring the Time to Deterioration for Health-Related Quality of Life in Patients With Metastatic Breast Cancer Using a Web-Based Monitoring Application: Longitudinal Cohort Study
Source: JMIR Cancer. 2021 Oct 12;7(4):e25776. doi: 10.2196/25776 (PMC8548964; doi:10.2196/25776)
Supplement: Multimedia Appendix 1 [file cancer_v7i4e25776_app1.docx]

# Appendix

**Table 1.** Number of complete questionnaires per visit.

| **Visit** | **Baseline** | **1** | **2** | **3** | **4** | **5** | **6** | **7** | **8** | **9** | **10** | **11** | **12** |
| --- | --- | --- | --- | --- | --- | --- | --- | --- | --- | --- | --- | --- | --- |
| **EQ-VAS** | 166 | 127 | 132 | 133 | 116 | 115 | 109 | 109 | 104 | 87 | 78 | 65 | 60 |
| **EQ-5D-5L** | 112 | 84 | 85 | 92 | 90 | 87 | 85 | 87 | 82 | 72 | 66 | 58 | 53 |
| **QLQ-C30** | 165 | NA | NA | NA | 115 | NA | NA | NA | 103 | 87 | 78 | 65 | 59 |

**Table 2.** Univariate Cox-regression analysis adjusted for age and progression.

|  | | | **EQ-VAS** |  | **EQ-5D-5L** |  | **EORTC** |  |
| --- | --- | --- | --- | --- | --- | --- | --- | --- |
| **Variable** | | | **Hazard ratio (95%-CI)** | ***P*-value** | **Hazard ratio (95%-CI)** | ***P*-value** | **Hazard ratio (95%-CI)** | ***P*-value** |
| **Metastasis** | | |  |  |  |  |  |  |
|  | Brain | | 1.39 (0.58-3.33) | 0.466 | 0.24 (0.03-1.83) | 0.17 | 0.93 (0.33-2.65) | 0.891 |
|  | Lymph nodes | | 1.16 (0.77-0.75-1.82) | 0.508 | 1.40 (0.78-2.52) | 0.253 | 0.87 (0.52-1.46) | 0.516 |
|  | Bone | | 0.82 (0.53-1.28) | 0.381 | 0.85 (0.47-1.56) | 0.606 | 1.14 (0.68-1.89) | 0.617 |
|  | Lung | | 1.08 (0.69-1.69) | 0.736 | 0.69 (0.37-1.31) | 0.253 | 0.92 (0.55-1.55) | 0.76 |
|  | Pleura | | 0.39 (0.17-0.89) | 0.026 | 0.46 (0.14-1.50) | 0.198 | 0.90  (0.43-1.91) | 0.786 |
|  | Liver | | 1.65 (1.04-2.60) | 0.032 | 1.81 (0.99-3.29) | 0.052 | 0.91 (0.54-1.52) | 0.713 |
|  | Peritoneum | | 0.93 (0.34-2.55) | 0.883 | 0.80 (0.19-3.3) | 0.754 | 1.23 (0.45-3.40) | 0.688 |
|  | Skin | | 0.57 (0.21-1.55) | 0.270 | 0.80  (0.25-2.58 | 0.707 | 0.58 (0.18-1.86) | 0.364 |
|  | Other | | 0.66 (0.32-1.39) | 0.273 | 0.67 (0.24-1.89) | 0.450 | 1.64 (0.84-3.17) | 0.145 |
| **Systemic Group** | | |  |  |  |  |  |  |
|  | | CDK4/6-inhibitors +/- endocrine therapy | Reference | Ref | Ref | Ref | Ref | Ref |
|  | | Chemotherapy | 2.07 (1.09-3.93) | 0.025 | 1.90 (0.78-4.64) | 0.16 | 0.82 (0.42-1.64) | 0.598 |
|  | | Endocrine therapy | 1.83 (0.81-4.13) | 0.143 | 1.55 (0.89-4.9) | 0.452 | 1.26 (0.56-2.81) | 0.574 |
|  | | HER2-targeted therapy | 1.66 (0.86-3.23) | 0.133 | 2.14 (0.95-5.16) | 0.09 | 0.71 (0.35-1.43) | 0.341 |


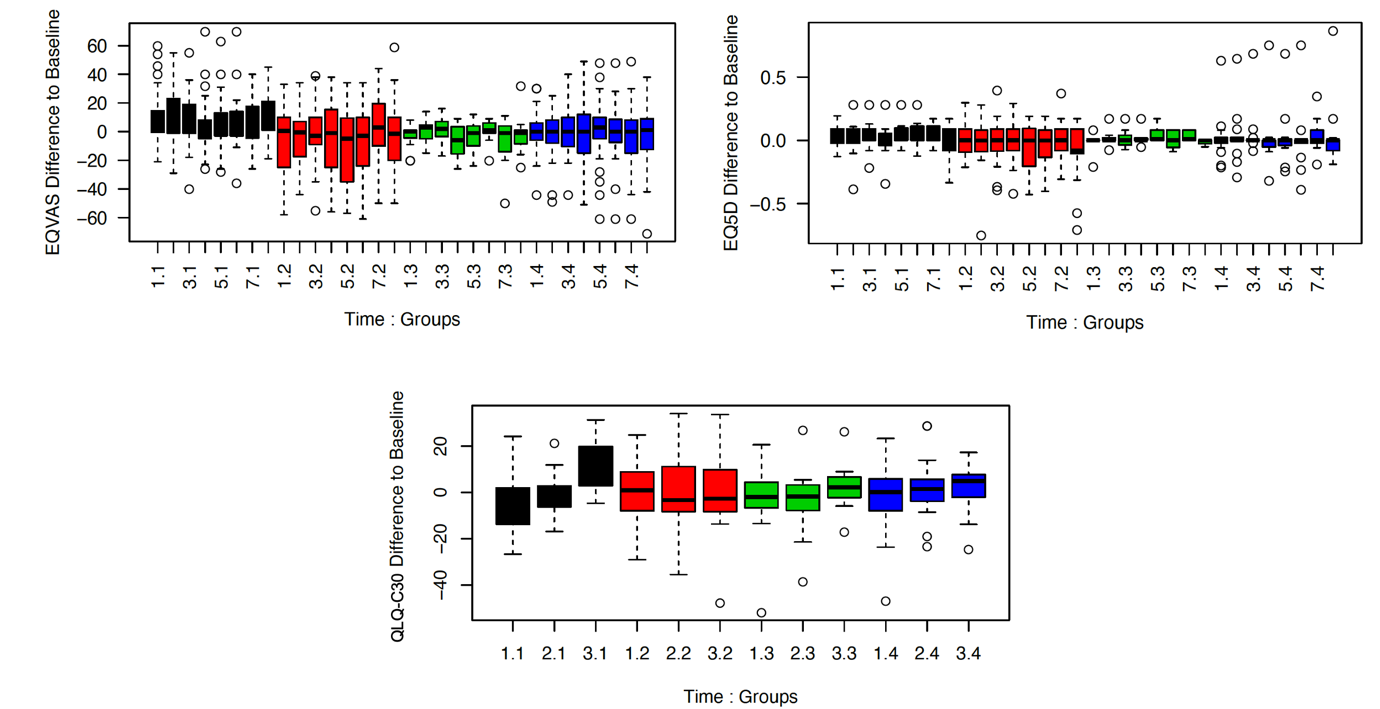


**Figure 1**. HRQoL score differences between the different visits and baseline for four systemic treatment groups.

Y-axis: Score difference to baseline, X-axis: encoded as [visit].[systemic treatment group]. Subgroups are further color-coded as follows: 1/black= CDK4/6 inhibitors+/- Endocrine therapy, 2/red=Chemotherapy, 3/green=Endocrine therapy, 4/blue=HER2-targeted therapy.
